# Comparison of flapless versus conventional flap implant placement on soft tissue health and prosthodontic outcomes

**DOI:** 10.6026/973206300213809

**Published:** 2025-10-31

**Authors:** Deblina Saha, Monika Singh

**Affiliations:** 1 Department of Periodontology and Oral Implantology, Kalinga Institute of Dental Sciences, Odisha, India; 2 Department of Dentistry, All India Institute of Medical Sciences, Gorakhpur, Uttar Pradesh, India

**Keywords:** Flapless, conventional flap, implant placement, soft tissue

## Abstract

The effects of flapless versus conventional flap implant placement on soft tissue health and prosthodontic outcomes is of interest. Hence, a total of 130 patients were randomly assigned to either the flapless (65 patients) or conventional flap (65 patients) group. The results showed that the flapless technique preserved soft tissue health more effectively, with lower probing depths and higher keratinized gingival width. Both groups demonstrated high implant success rates and similar prosthodontic outcomes. Thus, the flapless group also experienced fewer complications, faster healing, and lower pain scores, making it a viable alternative to conventional flap surgery.

## Background:

The dental implant surgeries have greatly relied on the surgical procedure employed and the success of soft tissue in the affected area, which involves healing and maintenance [1]. Traditionally, dental implants were placed by a flap procedure, in which a cut is made, thereby exposing the underlying bone as a way of making the implant precise [2]. Nevertheless, more recent strategy dubbed as flapless implant placement has emerged. With flapless placement, no area of the gum tissue is removed, but small holes are directed through the liner with a finger- or needle-like object placed in the holes moving back and forth to the implant [3]. It is considered to have such benefits as a minimized level of surgical trauma, enhanced rehabilitation and post-traumatic healing periods, and the best possible maintenance of the soft tissue. Soft tissue health is also critical in the success of dental implants over the long run [4]. Stable soft tissue surrounding the implant is healthier and facilitates aesthetic outcome, avoids exposure of the implant, and promotes the support of the prosthesis. It is also more applicable in preservation of the soft tissue where flapless techniques are more effective since the surrounding gum structures are less affected [5]. And, another reason why flapless implant placement can be supposedly beneficial is the reason that it can make the patient more comfortable and reduce his chances of encountering complications such as infection or delayed healing [6]. Conventionally, flap technique on the other hand provide the surgeon easier access to the implant site hence enabling the surgeon to control the insertion points of the implant and insert it to more exact points. This is particularly important where the quality and / or quantity of the bone are not perfect or where the implant is inserted in harder to access region of the mouth [7]. Although flap surgery has many advantages, it can cause more after-surgery pain and longer time of recuperation because the delicacy of the tissue is disturbed [8]. Therefore, it is of interest to determine the comparative effects of flapless versus conventional flap implant placement techniques on soft tissue health and prosthodontic outcomes.

## Methodology:

This study aims to compare the outcomes of flapless versus conventional flap implant placement techniques on soft tissue health and prosthodontic outcomes. A total of 130 patients who are eligible for dental implant placement will be included in the study. The patients will be selected from a pool of individuals attending the dental clinic for implant treatment, with criteria ensuring they meet the necessary requirements for either technique. The inclusion criteria will consist of adult patients aged 18-70 years who require a single or multiple dental implants in the edentulous areas of the maxilla or mandible. Exclusion criteria will include patients with systemic diseases that affect healing, such as uncontrolled diabetes, smoking history, insufficient bone volume, or those who require bone grafting procedures. The patients will be randomly divided into two groups: 65 patients in the flapless implant placement group and 65 patients in the conventional flap implant placement group. Randomization will ensure that both groups are comparable in terms of baseline characteristics. Before the procedure, all participants will undergo a comprehensive clinical and radiographic examination, including 3D cone beam computed tomography (CBCT) scans, to assess the bone structure and available tissue for implant placement. Detailed medical histories and preoperative blood tests will be performed to ensure patient eligibility. Additionally, baseline measurements of soft tissue health, including probing depth, keratinized gingival width, and clinical attachment level (CAL), will be recorded. For the flapless group, implants will be placed with no incision into the gingiva, using a flapless technique under local anesthesia. The implant site will be marked, and a small opening will be made through the soft tissue for implant placement, with minimal disruption to the surrounding gingival tissue. For the conventional flap group, a mucoperiosteal flap will be raised to expose the bone, followed by implant placement. The flap will be sutured back in place to cover the implant. Both groups will receive standardized postoperative care, including antibiotics, analgesics, and post-surgical instructions for optimal healing. Patients will be scheduled for follow-up visits at 1 week, 1 month, 3 months, and 6 months to monitor healing and soft tissue health. Clinical assessments, including measurement of the gingival index, bleeding index, probing depth, and the width of keratinized gingiva, will be taken at each follow-up visit. Prosthodontic outcomes will be assessed based on the success rate of implant placement, the quality and stability of the final prosthesis, and the aesthetic outcomes at the 6-month mark. The evaluation will also include the patient's subjective satisfaction with the final result, measured using a visual analogue scale (VAS) and a standardized questionnaire. The data will be analyzed using statistical software (SPSS or equivalent), with a significance level set at p<0.05. The primary outcomes (soft tissue health and prosthodontic outcomes) will be compared between the two groups using independent t-tests or chi-square tests, as appropriate. Multivariate analysis will be conducted to control for potential confounders.

## Results:

The numbers of the patients comprising the study were 130, out of which 65 patients participated in the flapless implant placement procedure and 65 were subjected to the conventional flap implant placement procedure. The demographic findings of the participants illustrated in Table 1 (see PDF) indicated that both the groups were matched on parameters of age, sex, smoking history and presence of any systemic condition. The sample was 45.6 years in age (standard deviation 12.0), and there was no significant variation between the two categories regarding the flap (flapless: 45.2+/-12.6, conventional flap: 46.1+/-11.4). The groups also had more or less equal gender distribution since there were 62 men and 68 women patients in total. Such parameters of soft tissue health as probing depth, keratinized gingival width, and clinical attachment level were placed in preoperative measurements as well. These baseline readings presented in Table 2 (see PDF) did not reveal any significant variation between the two groups to show that both groups were at par as far as soft tissue health was concerned before being subjected to implant placement. The flapless group had an additional probing depth of 2.2 mm (2.5), and the conventional flap group had an additional probing depth of 2.3 mm (2.6) followed by similar numbers in the keratinized gingival width and clinical attachment level. The health of the soft tissue assessments were evaluated one week following the implant placement as well as three months post the implant insertions. Table 3 (see PDF) actually reveals that at follow-up visit 1-week, the flapless group had significantly reduced probing depth (2.3 mm 0.4) while the conventional flap group showed rather large probing depth (2.7 mm 0.5). Also, the flapless group presented a bigger width of keratinized gingival (3.4 mm +/- 1.1) and a smaller bleeding index (0.5 +/- 0.3) when compared to the conventional flap group that had a width of 3.1 mm +/- 1.3 and a bleeding index of 0.7 +/- 0.4, respectively. The same table (Table 4 - see PDF) indicates that the flapless cohort also recorded improved soft tissue health at the 3 months follow-up. The probing depth was still notably less when using the flapless technique (2.1 mm +/- 0.3) than with conventional flap technique (2.5 mm +/- 0.4). In addition, the width of the keratinized gingiva was greater in the flapless group (3.6 mm +/- 1.0 vs. 3.2 mm +/- 1.2 within the conventional flap group) and the levels of clinical attachment were not bettered in either of the groups (1.2 +/- 0.7 vs. 1.3 +/- 0.8, correspondingly). The 6-month follow-up was used to measure the Prosthodontic outcomes. The implant success rates of the two groups were significant and there was no significant difference between the two methods. Table 5 (see PDF) indicates the implant success rate to be 98.5 and 97.0 in the flapless and conventional flap categories, respectively. Comparison of the aesthetic results and satisfaction with the results measured with the visual analog scales (VAS) between the participants was very close. The flapless technique was associated with a SLIGHTLY better aesthetic result (VAS score: 8.4 1.2 vs. 8.1 1.4 in the conventional flap group) and patient satisfaction (VAS score: 8.7 1.3 vs. 8.3 1.6), but not significantly. The flapless group had fewer issues in terms of the complication and healing time. In Table 6 (see PDF), the rate of infection rates in the force of one case (1.5%) and two cases (3.1%) were significantly different in the flapless group as compared to 3 (4.6%) and 4 (6.2%) cases of delayed healing in the conventional flap group. In addition, pain scores were significantly lower in flapless group (3.2 +/- 1.5) than in conventional flap group (4.5 + 1.8) indicating less painful recovery of the flapless technique. In general, the findings of the current study indicate that flapless implant placement is characterized by considerable benefits in the sphere of soft tissue maintenance and post-surgery comfort. Although the two methods led to good implant success rates and acceptable prosthodontic results, there were improved soft tissue health and less complication observed in flapless method. These results show that the flapless implant insertion might be a better option at least in regard to alleviating the pain of the patient and producing some superior results in soft tissue.

## Discussion:

Among the most important findings of this study, it could be stated that there was a better soft tissue health in the flap less procedure with lower probing depths and wider keratinized gingival width at 1-week and 3-month follow-up. This is in line with the studies by Elkhaweldi *et al.* (2015) [9], who concluded that the use of flapless implant insertions produces less tissue trauma, so the healing is quicker with more stable gingival architecture. On the same note, Tarpara *et al.* (2025) [10] noted that flapless placement is associated with the minimal gingival recession and increases the preservation of the peri-implant soft tissue. We add to these findings in that postoperative bleeding is less with the use of the flapless technique as well and this is especially significant when comparing the effects of reduced inflammation and healing. Nevertheless, there have been more mixed findings in other studies. As an example, Oliver *et al.* (2012) [11] discovered that although flapless placement of an implant did not compromise the preservation of the gingival tissue, it did not necessarily result in improved soft tissue results when issues related to bone grafting or complicated anatomies were concerned. This was not any shortcoming in our study because we excluded patients who needed bone grafts and this could be the reason why consistent beneficial results were recorded in the health of the soft tissues. Concerning the success of implant insertion and the prosthodontic outcome, the results of both groups in the present study indicate that the success rates were high (98.5 percent in the flapless and 97.0 percent in the conventional flap) with significant results not reached between the comparison groups. This can be correlated with the results of Chandrshekhar *et al.* (2024) [12] who concluded that implant placement in flapless mode leads to the same or, at times, even better implant success rates compared to typical flap procedures. It was also apparent in their study that flapless techniques lead to a minimized surgical trauma perhaps leading to a better biological milieu in which to heal but there was no statistically significant increase in the success rates between the methods. On the contrary, Van de Velde *et al.* (2008) [13] observed that the placement of flapless implants may cause in certain instances inadequate implant positioning whereby visibility is lower, which may impact the prosthodontic overall outcome. This did not constitute a problem in our research, where both groups showed strictness in positioning of the implants, and imaging (CBCT) in both the groups provided precise planning before surgery. Consequently, we found no major disparities in prosthodontic outcomes as a result of using the two methods, which are in line with the enumerated results by Jain *et al.* (2024) [14], which claimed that no difference occurs regarding impacting and aesthetic outcomes when one uses the flapless techniques. Our research also proved that flapless group had less post-operative complications like infection and slow healing process as well as pain score. These outcomes can be compared to those obtained by Tarpara *et al.* (2025) [10], who stated that compared to the traditional method of flaps surgery, flapless implant placement proved to cause much less postoperative discomfort and shorter healing time. The reason given to this is that flapless procedures involve less soft tissue manipulation thus producing less swelling, discomfort as well as pain during the healing process. Conversely, Bashutski *et al.* (2013) [15] noted that flapless surgery in any specific situation, and above all when performed by surgeons who are less experienced, may lead to complications, including altered placement of the implants or even the development of peri-implantitis. Nevertheless, the skill level and preoperative planning we had in our study, as well as the usage of advanced imaging and dedicated surgical approaches, were likely to reduce such risks, which contributed to the low complication rate of the study.

## Conclusion:

We show that flapless implant placement offers superior soft tissue health, fewer complications, and faster recovery compared to the conventional flap technique. Both approaches showed similar implant success and prosthodontic outcomes. Thus, flapless implant placement can be considered a viable and patient-friendly alternative in appropriate cases.
